# Effects of *Sparassis latifolia* neutral polysaccharide on immune activity *via* TLR4-mediated MyD88-dependent and independent signaling pathways in RAW264.7 macrophages

**DOI:** 10.3389/fnut.2022.994971

**Published:** 2022-09-14

**Authors:** Zening Qiao, Yue Zhao, Menghao Wang, Jinling Cao, Mingchang Chang, Shaojun Yun, Yanfen Cheng, Feier Cheng, Cuiping Feng

**Affiliations:** College of Food Science and Engineering, Shanxi Agricultural University, Jinzhong, China

**Keywords:** *Sparassis latifolia*, neutral polysaccharide, macrophage RAW264.7, immune activity, toll-like receptor-4, MyD88 signaling pathway

## Abstract

**Background:**

*Sparassis latifolia* (*S. latifolia*) is a precious edible fungus with multiple biological activities. To date, no study has been investigated the underlying molecular mechanism of immunoregulation caused by the neutral polysaccharide of *S. latifolia.*

**Materials and methods:**

To investigate immunomodulatory mechanism of *S. latifolia* neutral polysaccharide (SLNP), SLNP was obtained from *S. latifolia* and its structure, immune receptors and regulation mechanism were studied.

**Results:**

*S. latifolia* neutral polysaccharide consisted of arabinose, galactose, glucose, xylose, and mannose with a molar ratio of 6:12:63:10:5. SLNP was a pyran polysaccharide with a relative molecular weight of 3.2 × 10^5^ Da. SLNP promoted the proliferation of RAW264.7, which further induced the secretions of nitric oxide, TNF-α, IL-6, and IFN-β, and upregulated the immune receptor TLR4 expression. Moreover, SLNP increased remarkably the levels of TRAF6, IRF3, JNK, ERK, p38, and p38 mRNA and protein mediated by TLR4.

**Conclusion:**

*S. latifolia* neutral polysaccharide regulated the immune function of RAW264.7 through MyD88-dependent and -independent signaling pathways mediated by TLR4 receptor, which suggests that SLNP is a new immunomodulator.

## Introduction

*Sparassis latifolia* (*S. latifolia*) is a rare edible fungus and is generally called the cauliflower fungus in China owing to its shape. It has been reported that β-glucan content of *S. latifolia* exceeds 40% (1). Previous studies have shown that *S. latifolia* containing β-glucan has a variety of biological activities, such as improving immunity, lowering cholesterol, anti-diabetes, anti-cancer, and anti-inflammation and so on (2, 3). Nowadays, primary interest has been devoted to polysaccharides of *S. latifolia* because of their effectiveness in enhancing immune function (4). *S. latifolia* polysaccharide could enhance the response of hematopoiesis (5). The cyclophosphamide-induced leukemia mice are treated with *S. latifolia* polysaccharides orally, the recovery speed of leukocytes in the organs of the mice is accelerated, and the productions of interferon α (IFN-α), tumor necrosis factor α (TNF-α), and interleukin 6 (IL-6) and other cytokines are significantly increased (6). *S. latifolia* polysaccharides can exert antibacterial effects by inhibiting dysfunction of catabolism and energy metabolism (7). The novel acidic polysaccharides purified from *S. latifolia* in our previous study, with molecular weight (Mw) of approximately 215 Da–393 kDa, has a certain reducing ability and can scavenge effectively DPPH, -OH, O_2_^–^ free radicals and promote the proliferation of RAW264.7 macrophages (8).

As crucial immunocytes derived from differentiation of blood monocytes, macrophages are important components of the immune system and play vital roles in the innate immune response and adaptive immunity, which are one of the first lines of defense against the source of infections (9, 10). Activated macrophages can enhance the organism’s defense by phagocytosis and release of inflammatory mediators such as NO and pro-inflammatory cytokines including TNF-α and IL-6 (11). RAW264.7 is a macrophage/monocyte-like cell line used to investigate interactions of polysaccharides and macrophages (12–14).

Toll-like receptors (TLRs) are related to the activation of macrophages. TLR4 is a key pattern recognition receptor that recognizes pathogen-associated molecular patterns and submits signals to cytoplasm to activate transcription factors thus produce proinflammatory cytokines (15, 16). Recently, some studies have shown that many natural polysaccharides exert immunomodulatory functions by acting on TLR4 on the surface of macrophages, such as *Dendrobium officinale* polysaccharide and *Poria Cocos* polysaccharide exerted an immune-potentiating effect through TLR4 (17, 18). Once being recognized, TLR4 can trigger downstream signaling pathways and then medicate the activation of mitogen-activated protein kinase (MAPK), which is a critical signaling pathway and plays a crucial role in the immune system (19, 20). *S. latifolia* polysaccharides can act through the TLR4 receptor to enable the downstream MAPK signaling pathway to activate dendritic cells (21). *Platycodon grandiflorum* (PG) polysaccharides induce dendritic cell maturation by activating MAPK and NF-κB signaling downstream of TLR4 (22). TLR4 is a membrane receptor of *Pueraria lobata* polysaccharide (PLP), which induces functional maturation of dendritic cells through TLR4 signaling (23). As such, TLR4 and downstream signaling pathways can be the targets of polysaccharides. Although the immunomodulatory effect of acidic polysaccharides of *S. latifolia* has been reported in previous studies, whether the neutral polysaccharide of *S. latifolia* (SLNP) regulates the immune activity *via* TLR4-mediated downstream signaling pathways and the underlying mechanism is still unclear. Hence, it is vital to investigate the underlying molecular mechanism of immunoregulation caused by SLNP.

In this study, to investigate the immunomodulatory effect of SLNP and the underlying mechanism, we first extracted and purified a neutral polysaccharide from the *S. latifolia* fruiting body, the structure of which was characterized by a combination of chemical and instrumental analysis of monosaccharide composition, fourier transform infrared spectra (FT-IR), high performance liquid gel permeation chromatography (HPGPC), and nuclear magnetic resonance (NMR). In addition, we assessed the immune potential of SLNP in RAW264.7 macrophages, and investigated the relationship between the immune activity of SLNP and TLR4-mediated myD88 dependent and independent signaling pathways.

## Materials and methods

### Materials

Dry *S. latifolia* fruiting body were provided by Taihe edible fungus cultivation base in Shanxi Province of China, which were dried treatment in an oven at 35°C. The dry *S. latifolia* fruiting body were sliced into pieces, sieved into 200 mesh powder, and stored sealed at 4°C.

Macroporous resin HZ-830, DEAE-52 cellulose powder, 3-(4,5-dimethylthiazolyl-2)-2,5-diphenyl tetrazolium bromide (MTT), penicillin-streptomycin, dimethyl sulfoxide (DMSO), trypsin (Parenzyme), ethidium bromide, phenylmethyl sulfonyl fluoride (PMSF), and polyvinylidene difluoride (PVDF) were purchased from Solarbio Science & Technology Co. (Beijing, China). Dextran standard products of T-3, T-10, T-40, and T-70 were obtained from BioDee Biotechnology Co. (Beijing, China). Monosaccharide standards of D-glucose (Glc), D-mannose (Man), D-xylose (Xyl), D-galactose (Gal), L-arabinose (Ara), and D-fructose (Fru), and Lipopolysaccharide (LPS) were purchased from Sigma (St. Louis, MO, United States).

RAW264.7 cells were purchased from Cell Resource Center, Shanghai Institutes for Biological Sciences, Chinese Academy of Sciences (Shanghai, China). Fetal bovine serum (FBS) and Dulbecco’s modified eagle medium (DMEM) high glucose medium were obtained from Zhejiang Tianhang Biotechnology Co. (Hangzhou, China) and HyClone (Utah, United States). The Micro NO Content Assay Kit, SDS-PAGE gel configuration kit, BCA protein concentration determination kit, high-sensitivity chemiluminescence detection kit, and RIPA cell lysate were obtained from Beyotime Institute of Biotechnology (Shanghai, China). Assay kits for TNF-α, IL-6, and IFN-β were purchased from Shanghai Westang Biotechnology Co. (Shanghai, China). RNAiso Plus, SYBR^®^ Premix Ex TaqTM II (Tli RNaseH Plus), PrimeScriPt™ RT Master Mix (Perfect Real Time) were purchased from Takara Biomedical Technology Co., Ltd. (Dalian, China). The primary antibodies of TLR4 (bs-1021R), IRF3 (bs-2993R), JNK (bs-2592R), p-JNK (bs-1640R), ERK (bs-2637R), p38 (bs-0637R), and β-actin, and the secondary antibodies of HRP-labeled goat anti-mouse IgG (bs-0295G) were purchased from Bioss Biotechnology Co. (Beijing, China). And the primary antibodies of tumor necrosis factor receptor-related kinase 6 (TRAF6) (66498-1-lg) were purchased from Proteintech Group, Inc. (Wuhan, China).

### Extraction and purification of *Sparassis latifolia* neutral polysaccharide

The dry powder of SLNP was mixed with ultrapure water at a ratio of 1:40 (g:mL), and hydrolyzed at 75°C for 2 h. After being centrifuged at 4500 rpm for 10 min, the supernatant was concentrated, added ethanol at a ratio of 1:3 (mL:mL), and kept at 4°C for 12 h. The precipitate was collected by centrifugation at 5000 rpm for 5 min, and washed with acetone and ether for several times, dissolved in ultrapure water at 45°C for 8 h. Afterward, the supernatant was added Sevag solvent (Chloroform: N-butanol = 4:1), stirred by magnetic stirrer for 20 min, and centrifuged at 5000 rpm for 5 min. Next, the obtained supernatant was preliminarily decolorized and removed impurities by HZ-830 Macroporous Adsorption Resin to obtain crude polysaccharides. Then the 10 mg/mL crude polysaccharides were eluted in a DEAE-52 cellulose chromatography column (2.6 cm × 30 cm) with ultrapure water at a flow rate of 1 mL/min. The obtained polysaccharides were measured the content by the phenol sulfuric acid assay. Finally, the purified neutral polysaccharides were collected, dialyzed, and lyophilized.

### Molecular weight

The Mw of SLNP was evaluated by HPGPC (Agilent, United States) with a final concentration of 0.5 mg/mL and the injection volume of 20 μL using a detector differential refractive index detector and a TSK-GEL-4000 PWXL column (Agilent, United States) eluting with ultrapure water. The column temperature was 40°C, and the flow rate was 0.6 mL/min. T-dextran standards with different molecular masses were used for injection under the same conditions and the standard curve was drawn with the peak time as the abscissa and the relative Mw as the ordinate.

### Monosaccharide composition analysis

Twenty mg of SLNP was mixed with 4 mL of trifluoroacetic acid in a sealed test tube and was put into an oven to hydrolyze at 120°C for 6 h. Then the hydrolysate was added methanol to evaporate trifluoroacetic acid completely and dissolved in 1 mL of distilled water. Afterward, the obtained solution was diluted 10 times to measure the monosaccharide composition by ion chromatography (IC) with Dionex Carbopac PA10 column (250 mm × 4 mm) and Dionex pulsed amperometric detector (California, United States) with Au electrode. The detection conditions were as follows: the column temperature was 30°C, the injection volume was 25 μL, the flow rate was 0.45 mL/min, and elution mode was 10% 200 mmol/L NaOH and 90% ultrapure water. Glucose, fructose, mannose, xylose, galactose, and arabinose were selected as standard monosaccharide.

### Fourier transform infrared spectra analysis

The functional chemistry of SLNP (2 mg in KBr pellets) was detected with a Bruker Tensor 27 IR instrument (Karlsruhe, German) at a frequency range of 400–4000 cm^–1^.

### Nuclear magnetic resonance spectroscopy

*Sparassis latifolia* neutral polysaccharide was treated with deuterium (D_2_O, 99.9%) and lyophilized with D_2_O for three times to exchange protons. Afterward, SLNP was placed in a 5 mm NMR tube and dissolved in 0.5 mL of D_2_O. NMR-spectra was recorded with a Brucker AVANCE III 850 MHz NMR (Karlsruhe, Germany). The analysis included a 1D spectrogram (^1^H, ^13^C).

### Cell culture

The RAW264.7 macrophages were cultured in DMEM high glucose medium containing 10% fetal bovine serum in a 5% CO_2_ incubator at 37°C. Then the cells at the logarithmic growth phase and under stable growth conditions were selected for further experiments.

### Cell viability assay

The effects of SLNP on the viability of RAW264.7 cells were measured by MTT method. The RAW 264.7 macrophages were planted into 96-well plates at a density of 1 × 10^4^ cells per well and cultured overnight in a CO_2_ cell incubator. Next the cells were cultured with 100 μL of SLNP solution with 12 different concentrations between 1.95 μg/mL and 4000 μg/mL for 24 h, respectively, during which LPS (1 μg/mL) was used as a positive control group, and the culture medium was used as a negative control group. There were 6 replicates in each group. After that, cells in each well were added 20 μL MTT solution to continuously incubate in dark for 4 h. After discarding MTT and adding 150 μL of Dimethyl sulfoxide (DMSO) in each well, the cells were gently shaken on the shaker for 15 min and the cell viability was determined with a SpectraMax i3X microplate reader (Sunnyvale, United States) at 490 nm.

### Assay for NO and cytokine secretion

The RAW264.7 cells (2.5 × 10^5^ cells/well) under good growth conditions were seeded in 24-well plates and cultured overnight, and the old medium was discarded after adherent. Then the cells were treated with various concentrations of SLNP (62.5, 125, 250, 500 μg/mL), LPS (1 μg/mL), and the medium for 24 h. There were 4 replicates in each group. Next the supernatants in each well were collected and centrifuged to determine the levels of NO and IL-6, TNF-α, IFN-β by Micro NO Content Assay Kit and the corresponding ELISA kits.

To further study the effects of TLR4 antibody on the secretion of NO and cytokines, the RAW264.7 cells (2.5 × 10^5^ cells/well) in each well were treated with 20 μg/mL of TLR4 antibody for 1 h. Afterward, the cells were treated with 250 μg/mL of SLNP, 1 μg/mL of LPS, and the medium for 24 h. Each group had 4 replicates. After incubation, the supernatants were collected to measure the concentrations of NO and cytokines of IL-6, TNF-α, IFN-β by Micro NO Content Assay Kit and the corresponding ELISA kits.

### Quantitative analysis of cytokine mRNA expression

The RAW264.7 cells were planted into 6-well plates at a density of 2 × 10^6^ cells per well for 12 h. After discarding the old medium, cells were incubated with 125, 250, 500 μg/mL of SLNP, 1 μg/mL of LPS, and new medium for another 24 h, respectively. Total RNA was extracted from RAW264.7 cells using the RNAiso Plus according to the manufacturer’s protocols. After that, the purity and content of RNA were measured by Nanodrop 2000c (Thermo Fisher, Delaware, United States). Next, cDNA was synthesized from the total RNA with the PrimeScript^®^ RT reagent Kit. Afterward, the mRNA expression levels of *TLR4*, *TRAF6*, *IRF3*, *JNK*, *ERK*, *p38*, and β*-actin* in RAW264.7 macrophages were detected by real-time fluorescent quantitative polymerase chain reaction (qPCR) under the following conditions: 95°C for 90 s, 40 cycles of 95°C for 5 s, 60°C for 30 s, 72°C for 30 s, 95°C for 15 s, 60°C for 1 min, and 95°C for 15 s. The levels of the target genes were calculated according to 2^–ΔΔCt^ method with the β-actin gene as the internal reference. The primers used for qPCR were listed in Table 1.

**TABLE 1:** Primers used for qPCR.

No.	Genes	Primers (5′- > 3′)	Primer location (start)	Product size (bp)	Genbank No.
1	β-actin	AGCCATGTACGTAGCCATCC CTCTCAGCTGTGGTGGTGAA	1063 1145	83	NM_007393.3
2	TLR4	AATCTGGTGGCTGTGGAG CCCTGAAAGGCTTGGTCT	1536 1766	147	NM_021297.2
3	TRAF6	AGGGCTACGATGTGGAGTT TTTACCGTCAGGGAAAGAAT	162 396	235	NM_009424.3
4	IRF3	CGCTACACTCTGTGGTTCTG GATAGGCTGGCTGTTGGA	1000 1179	180	NM_016849.4
5	JNK	ATTGAACAGCTCGGAACAAC GAGTCAGCTGGGAAAAGCAC	984 1123	140	NM_013693.2
6	ERK	TGACCTCAAGCCTTCCAACC ATCTGGATCTGCAACACGGG	683 770	88	NM_011949.3
7	p38	TCACGCCAAAAGGACCTACC ATTCCTCCAGTGACCTTGCG	632 738	107	NM_001168514.1

### Western blot analysis

After treating with different concentrations of SLNP (125, 250, and 500 μg/mL), 1 μg/mL of LPS and new medium for 24 h, the RAW264.7 cells in each well were added 150 μL of RIPA cell lysate containing PMSF and protein phosphatase inhibitor to lyse for 30 min. After centrifugation at 12,000 r/min for 15 min, the supernatants were collected and stored at −80°C for further use. Afterward, the total protein was quantified using the BCA method, subjected to SDS-PAGE, and transferred onto PVDF membranes. Next, the PVDF membranes were blocked with 5% non-fat milk at room temperature for 2 h, added the specific primary antibody of TLR4 (diluted 1:500), β-actin (1:1000), IRF3 (1:1000), JNK (1:1000), ERK (1:1000), p38 (1:1000), P-JNK (1:1000), and TRAF6 (1:1000), respectively, and shaken overnight at 4°C. After being rinsed 3 times with TBST, the PVDF membrane was added HRP-labeled goat anti-mouse IgG (diluted 1:3,000) and shaken at room temperature for 2 h. After the PVDF membrane was washed with TBST, the antibody-specific protein was observed in a fluorescence imager using eECL kit.

### Statistical analysis

The results were expressed as the mean ± standard error (SE). Graphpad Prism 5.0 software was used for one-way analysis of variance followed by Duncan’s multiple comparisons. *P* < 0.05 was regarded as statistically significant and *P* < 0.01 was considered to indicate a statistically highly significant.

## Results

### Isolation and purification of *Sparassis latifolia* neutral polysaccharide

**FIGURE 1:**
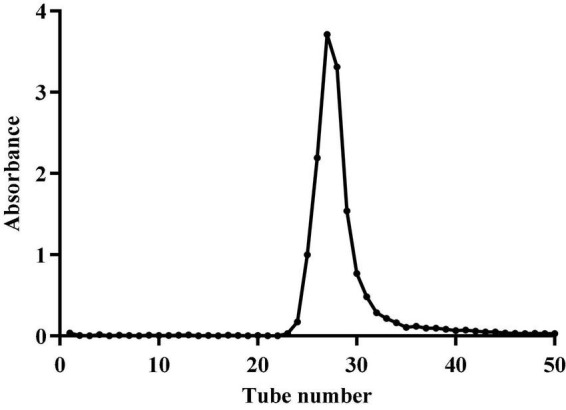
Elution curve of crude polysaccharide from *Sparassis latifolia* on DEAE-52 cellulose column.

**FIGURE 2:**
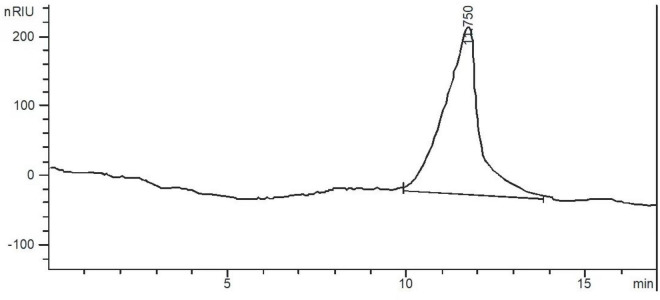
High performance gel permeation chromatogram (HPGPC) of *Sparassis latifolia* neutral polysaccharides SLNP.

After the crude polysaccharides were separated by a DEAE-52 cellulose column, the elution curve was a single and symmetrical peak (Figure 1). Next, the eluate was collected, concentrated, dialyzed, and freeze-dried to obtain light yellow neutral polysaccharides. Then the homogeneous SLNP were obtained after being collected and frozen-drying. The HPGPC of SLNP (Figure 2) showed that this fraction had a single and symmetrical peak and the relative Mw was 3.2 × 10^5^ Da.

### Preliminary structural characterization of *Sparassis latifolia* neutral polysaccharide

**FIGURE 3:**
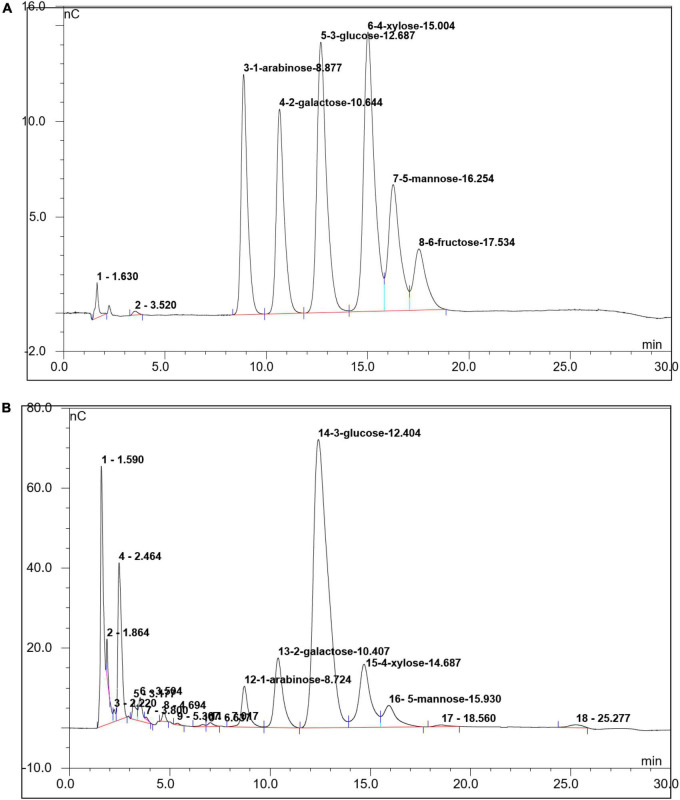
Ion chromatogram of *Sparassis latifolia* neutral polysaccharides SLNP. **(A)** Standard Sample; **(B)** SLNP.

The IC technology was used to determine the monosaccharide composition of SLNP. As shown in Figure 3, SLNP was mainly composed of arabinose, galactose, glucose, xylose and mannose with molar ratio of 6:12:63:10:5, respectively. Galacturonic acid and glucuronic acid were not detected, which indicated that SLNP was a neutral polysaccharide (24).

**FIGURE 4:**
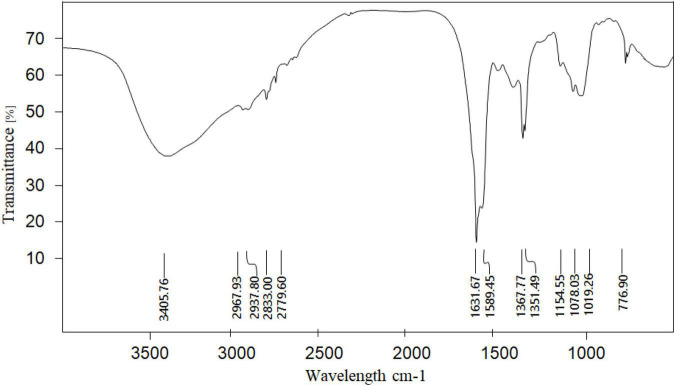
Fourier infrared spectrum of *Sparassis latifolia* neutral polysaccharides SLNP.

Fourier transform infrared spectroscopy was used to analyze the functional group structure of the SLNP. As shown in Figure 4, the absorption peak at 3406 cm^–1^ was caused by the stretching vibration of the hydrangea polysaccharide molecule -OH, the absorption peak in the range of 2780–2968 cm^–1^ was caused by the stretching vibration of C-H in the polysaccharide structure (25, 26), the absorption peak at 1631 cm^–1^ was the flexural vibration absorption peak of -OH, and the absorption peak near 1367 cm^–1^ was the flexural vibration absorption peak of C-H (27, 28). The absorption peaks at 1154–1019 cm^–1^ were the absorption peaks caused by the stretching vibration of the C-O-C structure and the C-O-H variable-angle vibration on the polysaccharide ring of *S. latifolia*, 1078 and 1019 cm^–1^ were the characteristic absorption of the pyran ring (29). There was the characteristic absorption peak of C-H variable angle vibration of α-type glycosidic bond near 840 cm^–1^, and the absorption peak near 890 cm^–1^ was the characteristic absorption peak of C-H variable angle vibration of β-type glycosidic bond, indicating the existence of a pyranose ring connected by α-glycosidic bond and β-glycosidic bond (30). In addition, no absorption peak was found near 1730 cm^–1^, indicating that there is no uronic acid in SLNP (31).

**FIGURE 5:**
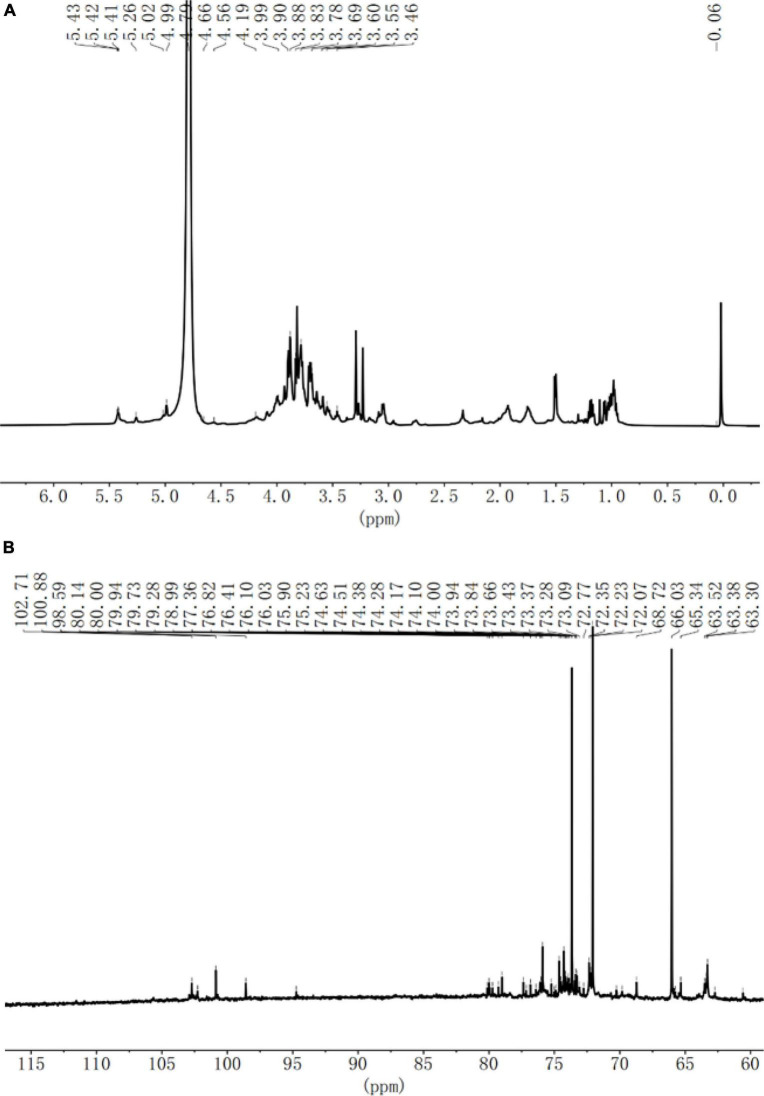
**(A)** 1H NMR spectra of *Sparassis latifolia* neutral polysaccharides SLNP. **(B)** (C)C^13^ NMR spectra of SLNP.

Nuclear magnetic resonance was used to analyze the glycosidic bond configuration of the SLNP. As shown in Figures 5A,B, in the ^1^H-NMR spectrum, the chemical shift at 3.5–4.5 ppm was the proton peak of the sugar ring carbon, and there was an anomeric proton signal absorption peak in the range of 4.5–5.5 ppm. Specifically, the chemical shift of 5.0–5.5 ppm indicates that the polysaccharide is mainly in the configuration, and the weak peak at 4.5–4.6 ppm indicates that the polysaccharide structure has a small amount of β configuration. In the anomeric carbon region of the ^13^C-NMR spectrum, the two absorption peaks of 98.6 and 100.8 ppm were α-type end group carbon signals, and the absorption peaks of 102.3, 102.7, and 102.9 ppm were β-type. The end-group carbon signal, of which the absorption peak signal at 100.8 ppm was stronger, and the signal at 98.6, 102.3, 102.7, and 102.9 ppm were relatively weak, indicating that SLNP is mainly composed of five monosaccharide residues. Consistent with the results of monosaccharide composition, the main chain might consist of one α-pyranose residue, one α-type sugar residue and three β-type sugar residues to form the side chain. The chemical shifts of 70.3–78.5 ppm were assigned to the unsubstituted C_2_-C_5_ structure on the sugar ring.

### Effects of *Sparassis latifolia* neutral polysaccharide on cell viability of RAW264.7 cells

**FIGURE 6:**
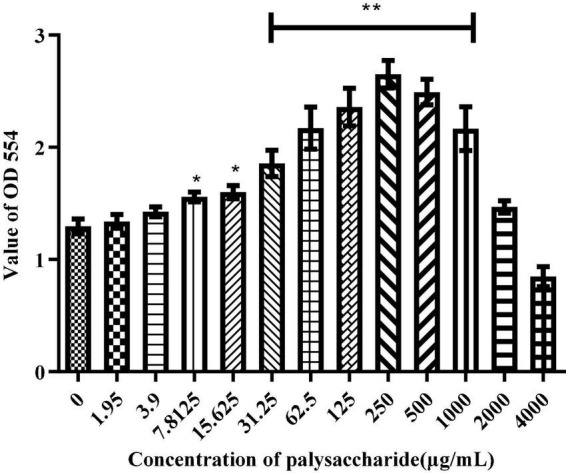
Effects of *Sparassis latifolia* neutral polysaccharides SLNP on RAW 264.7 macrophage viabilities. *p < 0.05, **p < 0.01 vs. control group.

The RAW264.7 macrophages were treated with different concentration of SLNP for 24 h to determine the cell viability by MTT assay. As shown in Figure 6, the proliferation ability of RAW264.7 cells were increased first and then decreased with the increase of SLNP concentration. The proliferation ability was significantly up-regulated after treated with SLNP (7.8125–1000 μg/mL) compared to the control group (*P* < 0.05). Moreover, 250 μg/mL of SLNP had the strongest ability to promote the proliferation of RAW264.7 cells and the upregulation rates were 103.84%. However, after SLNP concentration exceeded 250 μg/mL, the proliferation activity of RAW264.7 cells gradually decreased, and when the concentration was 4,000 μg/mL, SLNP strongly inhibited the proliferation activity of RAW264.7 cells with the inhibition rate of 34.54%.

### Effects of *Sparassis latifolia* neutral polysaccharide on production of NO and cytokines in RAW264.7 cells

**FIGURE 7:**
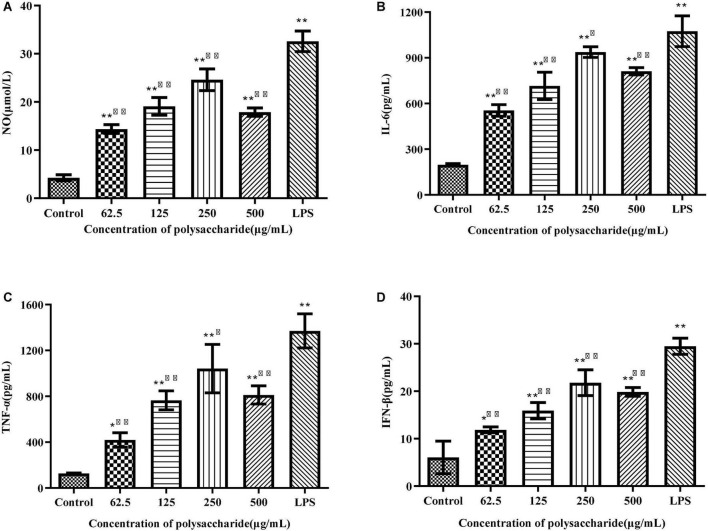
Effects of SLNP on NO **(A)**, IL-6 **(B)**, TNF-α **(C)**, IFN-β **(D)** production in RAW264.7 macrophage. Compared with blank control group, *p < *0.05*,**p < *0.01;* Compared with LPS control group, ^Δ^*p* < *0.05*, ^ΔΔ^*p* < *0.01.*

Activated macrophages can secrete a series of chemokines and cytokines, which play important roles in activating adaptive immune responses and regulating other immune responses (32). ELISA was used to detect the production of NO, IL-6, TNF-α, and IFN-β. Compared with the control group, different concentrations of SLNP up-regulated significantly the levels of NO (Figure 7A), IL-6 (Figure 7B), TNF-α (Figure 7C), and IFN-β (Figure 7D) in RWA 264.7 macrophages (*P* < 0.01), of which 250 μg/mL of SLNP had the strongest promotion ability and the upregulation rates were 477.98, 375.45, 721.52, and 260.88%, respectively. Meanwhile, LPS could promote remarkably RWA 264.7 macrophages to produce NO, IL-6, TNF-α, and IFN-β (*P* < 0.01). Above results indicate that SLNP might display immune enhancing activity by inducing the production of NO and cytokines in RAW264.7 cells.

### Participation of TLR4 in *Sparassis latifolia* neutral polysaccharide-induced macrophages activation

**FIGURE 8:**
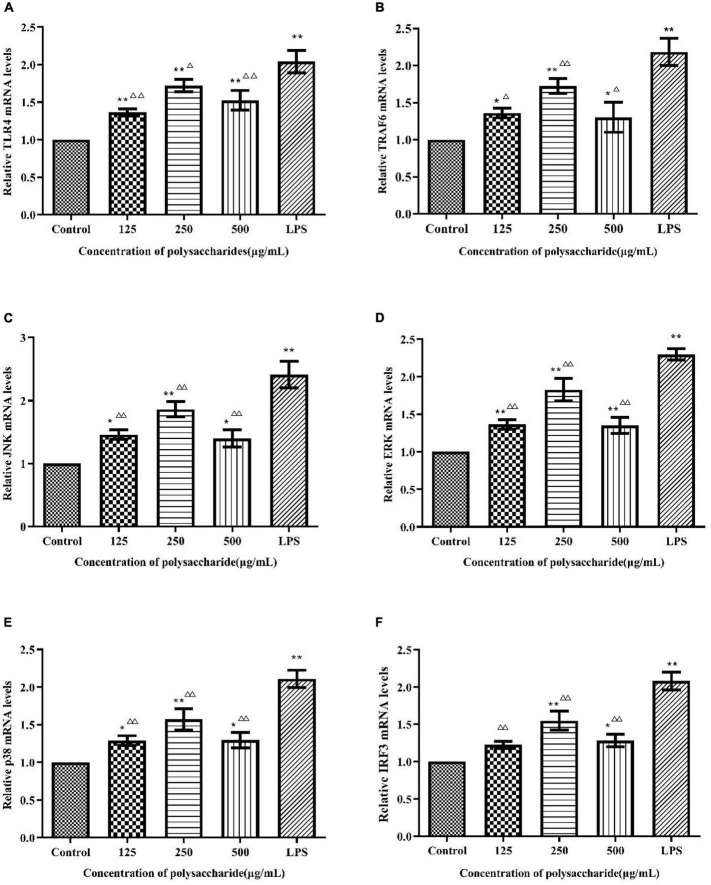
Effects of *Sparassis latifolia* neutral polysaccharides SLNP on mRNA expression of TLR4 **(A)**, TRAF6 **(B)**, JNK **(C)**, ERK **(D)**, p38 **(E)**, and IRF3 **(F)** on RAW264.7 macrophage. Compared with blank control group, *p < *0.05*, **p < *0.01;* Compared with LPS control group, ^Δ^*p* < *0.05*, ^ΔΔ^*p* < *0.01.*

**FIGURE 9:**
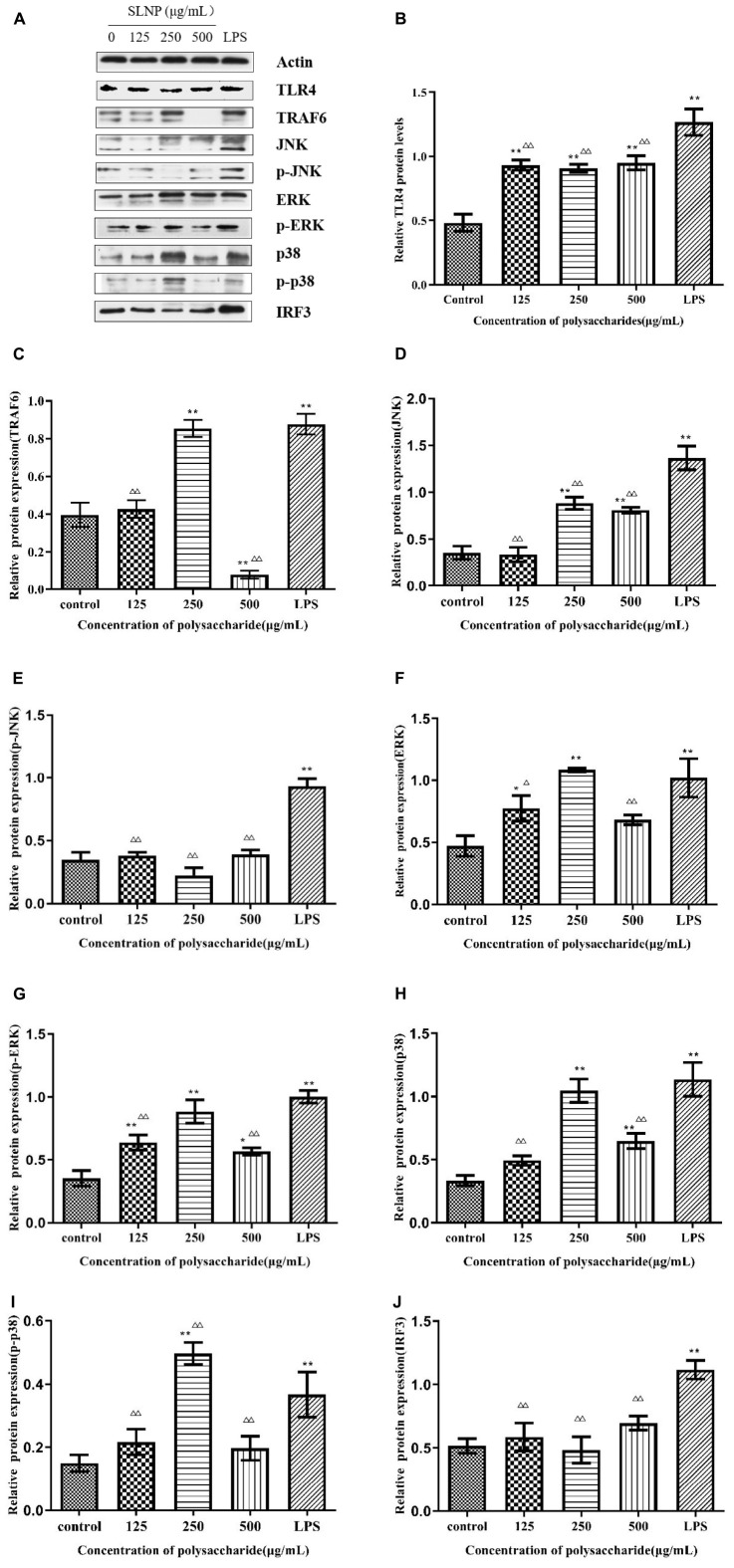
Effects of *Sparassis latifolia* neutral polysaccharides SLNP western blot results on protein expression. **(A)**, TLR4 **(B)**, TRAF6 **(C)**, JNK **(D)**, p-JNK **(E)**, ERK **(F)**, p-ERK **(G)**, p38 **(H)**, p-p38 **(I)**, and IRF3 **(J)** in RAW264.7 macrophage. Compared with blank control group, *p < *0.05*, **p < *0.01;* Compared with LPS control group, ^Δ^*p* < *0.05*, ^ΔΔ^*p* < *0.01.*

**FIGURE 10:**
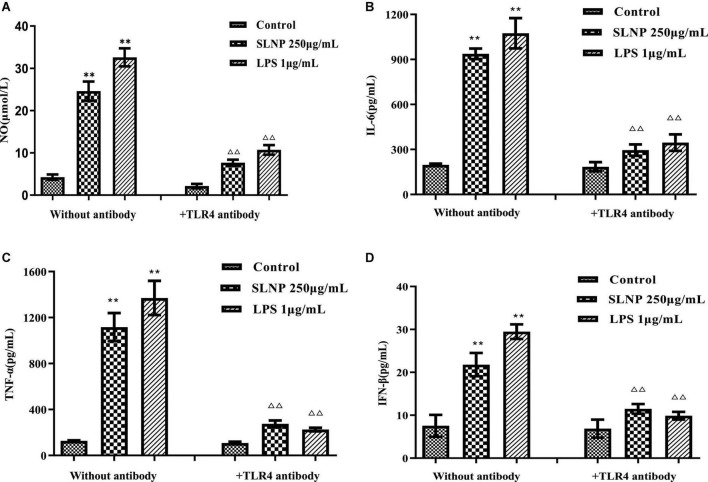
Effects of TLR4 antibodies on NO **(A)**, IL-6 **(B)**, TNF-α **(C)**, and IFN-β **(D)** production in RAW264.7 macrophage induced by *Sparassis latifolia* neutral polysaccharides SLNP. Compared with blank control group, *p < *0.05*, **p < *0.01;* Compared with LPS control group, ^Δ^*p* < *0.05*, ^ΔΔ^*p* < *0.01.*

To verify whether TLR4 has participated in SLNP-induced macrophages activation, we investigated the expression of TLR4 mRNA and protein, and the effects of TLR4 antibody on the secretion of NO and cytokines induced by SLNP. The results showed that compared with the control group, the expression levels of TLR4 mRNA (Figure 8A) and protein (Figure 9B) in macrophages were significantly elevated after treatment with different doses of SLNP and 1 μg/mL of LPS (*P* < 0.01). And treatment with 500 μg/mL SLNP increased markedly TLR4 mRNA and protein levels by 52.33 and 96.82%, respectively, implying that TLR4 is an immune recognition receptor in which SLNP plays an immunomodulatory role. However, blocking TLR4 signaling with the specific TLR4 antibody reversed the elevation of NO and cytokines in SLNP-induced RAW264.7 macrophages. As shown in Figure 10, the levels of NO (Figure 10A), IL-6 (Figure 10B), TNF-α (Figure 10C), and IFN-β (Figure 10D) were remarkably increased in macrophages RAW264.7 treated with 250 μg/mL of SLNP or LPS compared with the control group (*P* < 0.01). However, the TLR4 antibody decreased significantly the levels of NO (Figure 10A), IL-6 (Figure 10B), TNF-α (Figure 10C), and IFN-β (Figure 10D) by 68.87, 68.45, 75.38, and 47.25% compared with 250 μg/mL of SLNP group. The similar trends were found in RAW264.7 cells induced by 1 μg/mL of LPS. In short, these results illustrate that TLR4 participates in SLNP-induced macrophage activation.

### *Sparassis latifolia* neutral polysaccharide activated MyD88-dependent signaling pathway in RAW264.7 cells

In order to explore whether the SLNP-induced up-regulation of TLR4 caused the activation of MAPK signaling pathway, the cells were treated with different concentrations of SLNP, and the mRNA levels of *TRAF6*, *JNK*, *ERK*, and *p38* were measured by qPCR. Meanwhile, to further clarify the mechanism by which SLNP activates the MyD88-dependent pathway, the total levels of TRAF6 and phosphorylation levels of JNK, ERK and p38 were measured by Western Blot (Figure 9A).

As shown in Figure 8, different concentrations of SLNP significantly up-regulated the mRNA levels of *TRAF6* (Figure 8B), *JNK* (Figure 8C), *ERK* (Figure 8D), and *p38* (Figure 8E) compared with the control group (*P* < 0.05). Treatment with 250 μg/mL of SLNP elevated remarkably these indexes by 72.70, 86.54, 82.77, and 56.98%, respectively. The similar trends were found in the protein levels of TRAF6 (Figure 9C), JNK (Figures 9D,E), ERK (Figures 9F,G) and p38 (Figures 9H,I). Compared with the control group, the relative protein levels of TRAF6 (Figure 9C), JNK (Figures 9D,E), ERK (Figures 9E,G) and p38 (Figures 9H,I) in RAW264.7 cells were significantly up-regulated in 250 μg/mL of SLNP group, with the up-regulation rate of 115.13, 147.66, 131.21, and 214.01%. These results suggest that SLNP exerts the immunomodulatory effects by activating MyD88-dependent pathway and the downstream MAPK signaling pathway.

### *Sparassis latifolia* neutral polysaccharide activated MyD88-independent signaling pathway in RAW264.7 cells

TLR4 initiates MyD88-independent signaling pathways which increase the expression of IRF3 resulting in the expression of IFN-β (33). As shown in Figure 8F, after being incubated with SLNP, the expression of *IRF3* mRNA in RAW264.7 cells was increased significantly compared with the control group. The expression of IRF3 protein also showed an increasing trend after treated with SLNP (Figure 9J). Treatment with 500 μg/mL of SLNP increased remarkably the levels of IRF3 mRNA and protein by 28.13 and 18.00% in the RAW264.7 cells, respectively. These results indicate that SLNP activates the MyD88-independent signaling pathway.

## Discussion

The fungal polysaccharides have long been believed to possess benign immunoregulatory effects with low toxicity. Furthermore, they are considered potent immunomodulatory agents since they activate both innate and adaptive immune responses (34). In addition, polysaccharides can stimulate the secretion of immune factors while activating innate immunity (35, 36). The immunological activity of polysaccharide is strongly associated with its structure, such as Mw, monosaccharide composition and glycosidic bonds (37–39). In this study, a homogeneous polysaccharide with immunological activity was isolated and purified from *S. latifolia* with the relative Mw of 3.2 Da × 10^5^ Da. Moreover, previous reports have demonstrated that polysaccharides containing galactose, glucose, arabinose and mannose might have tight association with the immunomodulatory activity (40). Similar results were obtained in our study which found that SLNP consisted of arabinose, galactose, glucose, xylose and mannose at the molar ratio of 6:12:63:10:5, respectively. The high proportion of galactose and glucose in SLNP exert a strong immune activity. The FT-IR demonstrated that SLNP had strong absorption peaks near 3400, 2900, and 1600 cm^–1^, indicating that SLNP has the characteristic functional group structure of polysaccharides (41, 42). In addition, the single symmetrical peak presented by the main peak of SLNP indicates that it is a homogeneous polysaccharide. In summary, the immunomodulatory effect of SLNP is tightly related with its structure. However, the structure of SLNP is not fully clarified. Further study will be conducted to explore its structure deeply.

Macrophages are the main components of the mononuclear phagocyte system, which can not only initiate innate immune response, but also participate in cellular immune responses. The activated macrophages can phagocytose pathogenic microorganisms, process and present antigens, and simultaneously synthesize and secrete chemokines and cytokines to enhance the body’s immune defense capabilities (43–45). A great deal of natural polysaccharides can produce an immune effect primarily *via* macrophages (46, 47). *Morchella sextelata* polysaccharides with a concentration of 50–400 μg/mL can promote the proliferation of RAW264.7 cells (48). Polysaccharides isolated from *Sarcodon aspratus* are not toxic to RAW264.7 cells within a certain concentration range of 25–100 μg/mL (49). *Russula alutacea Fr.* polysaccharides exhibit the capacity to promote the proliferation of RAW264.7 cells at 25–200 μg/mL, and don’t show cytotoxicity even when the concentration reaches 600 μg/mL (50). These results support our study that SLNP not exceeding 1000 μg/mL was able to promote the proliferation of macrophage RAW264.7, indicating that SLNP within 1000 μg/mL don’t have cytotoxic effect on RAW264.7 cells. Hence, the dose used in the following studies was based on these results. Moreover, different concentrations of SLNP increased obviously the secretion of NO, IL-6, TNF-α, and IFN-β, suggesting that SLNP can stimulate macrophages to improve immunity. Similar result is obtained in a previous study of polysaccharides isolated from *Ophiocordyceps sinensis* mycelia (OSP), which shows that OSP significantly improves the immunomodulatory activity in macrophage RAW264.7 cells by promoting the production of TNF-α, IL-6, and IL-1β of macrophage RAW264.7 cells (51). However, when the concentration of polysaccharides exceeds 1000 μg/mL, the phenomenon of inhibition appears. Similar results are obtained in a previous study that a pectic polysaccharide from *Cucurbita moschata Duch* exerts significant suppressive effects on macrophages at 500 μg/mL (*P* < 0.05) (52). The suppression effects of polysaccharides on macrophages may be caused by the facts that high concentrations of polysaccharides may have caused mitochondrial dysfunction, especially the loss of transmembrane mitochondrial potential (53).

The immunomodulatory activity of fungal polysaccharides is inseparable from the immune receptors of macrophages. Polysaccharides bind to immune receptors to activate downstream signal transduction pathways by receptor mediation, transmit signals into cells, initiate immune responses, and promote downstream cytokine secretion (54). TLR4 is a pattern recognition receptor and usually expresses in immune cells including macrophages. The TLR4 signaling pathway is normally regarded to play a vital role in the activation of immune cells (55). Previous studies have shown that TLR4 is a key and undisputed target point of polysaccharides for macrophages, such as *Sarcodon aspratus* polysaccharide (49), *Coriolus versicolor* polysaccharide (56), *Polyporus umbellatus* polysaccharides (57). In the present study, SLNP could promote the mRNA and protein levels of receptor TLR4, and TLR4 antibody blocked the effect of SLNP to stimulate macrophages to secrete NO, IL-6, TNF-α, and IFN-β, which shows that TLR4 is one of the immune receptors of SLNP and involved in the SLNP-mediated activation of macrophages.

TLR4 mediates the secretion of TNF-α and IL-6 through the MyD88 signaling pathway (58). TLR4 can activate two different signaling networks, the MyD88-dependent and MyD88-independent signaling pathways (59). Once antigen is recognized by TLR4, the activated TLR4 recruit adapter protein MyD88 to induce the subsequent response. MyD88-dependent signaling pathways can activate MAPK/NF-κB and thus cause the secretion of cytokines (60). Specifically, the activated TLR4 can promote the phosphorylation of interleukin-1 receptor-related kinase (IRAK-1) to activate TRAF6 (61). Further, the MAKPs family (including JNK, ERK, and p38) is activated to produce phosphorylation and promote the secretion of downstream related immune cytokines (62). In this study, SLNP could promote TLR4 expression, upregulated the mRNA expression of TRAF6, IRF3, JNK, ERK and p38, the protein expression of TRAF6, IRF3, p-JNK, p-ERK, and p-p38, which was proved by the study of JCH-1, a purified polysaccharide isolated from *Isaria cicadae Miquel*. JCH-1 could promote TLR4 expression and up-regulated ERK, JNK, p38 phosphorylation, which indicated that JCH-1 activated RAW264.7 cells through TLR4-MAPK signaling pathway (59). These results imply that SLNP can promote the secretion and expression of various immune cells in the nucleus, such as TNF-α, IL-6, etc., by activating MyD88-dependent signaling pathways *via* TLR4, prompting the activation of TRAF6 to further activate the three target points of JNK, ERK and p38 in the MAPK signaling pathway. In addition, TRIF, another adaptor molecule of TLR4, initiates MyD88-independent signaling pathways resulting in the delayed activation of NFκB. TRIF also phosphorylate IRF3 resulting in the expression of IFN-β (63). Our results indicate that SLNP binding to TLR4 receptor can also signal through MyD88-independent pathways to activate IRF3 and catalyze the expression of IFN-β.

## Conclusion

After decolorization and impurity removal by HZ-830 macroporous resin and DEAE-52 separation, the *S. latifolia* polysaccharide was purified by Sepharose CL-6B to obtain the SLNP with the relative Mw of 3.2 Da× 10^5^ Da. SLNP was a pyran polysaccharide composed of glucose and galactose. SLNP showed a single symmetrical peak, with an excellent separation effect, high purity, and homogeneity. SLNP could promote the proliferation of RAW264.7 macrophages, which further induced the increased concentration of NO, TNF-α, IL-6, and IFN-β. However, the TLR4 antibody could inhibit significantly the secretion of NO, IL-6, TNF-α, and IFN-β. What’s more, SLNP increased remarkably the mRNA and protein levels of TLR4 receptor and the relative expression of mRNA and protein of the signal transduction pathway-related genes TRAF6, IRF3, JNK, ERK, p38, and p38 mediated by the immune receptor TLR4 on the surface of macrophages RAW264.7. These results indicate that TLR4 is the receptor of SLNP and can regulate the immune function of macrophage RAW264.7 through the MyD88-dependent and -independent signaling pathways mediated by the TLR4 receptor.

## Data availability statement

The datasets presented in this study can be found in online repositories. The names of the repository/repositories and accession number(s) can be found in the article/supplementary material.

## Author contributions

CF conceived and designed the experiments. ZQ, YZ, and MW coordinated the experiments, contributed to data interpretation, and manuscript writing. JC, MC, SY, YC, and FC participated writing – review and editing. All authors read and approved the final manuscript.

## Abbreviations

SLNP: *Sparassis latifolia* neutral polysaccharide
DMSO: dimethyl sulfoxide
PMSF: phenylmethyl sulfonyl fluoride
PVDF: polyvinylidene difluoride
Glc: D-glucose
Man: D-mannose
Xyl: D-xylose
Gal: D-galactose
Ara: L-arabinose
Fru: D-fructose
LPS: Lipopolysaccharide
IC: Ion chromatography
FT-IR: Fourier transform infrared spectroscopy
FBS: fetal bovine serum
MTT, [3-(4: 5-dimethylthiazol-2-yl)-2,5-diphenyl tetrazolium bromide]
MW: molecular weights
HPGPC: high performance gel permeation chromatography
PMSF: phenylmethylsulfonyl fluoride
ECL: enhanced chemiluminescence.
